# Recent advances in understanding, diagnosing and treating venous thrombosis

**DOI:** 10.12688/f1000research.27115.1

**Published:** 2020-10-06

**Authors:** Noel C Chan, Jeffrey I Weitz

**Affiliations:** 1 Thrombosis and Atherosclerosis Research Institute and McMaster University, Hamilton, Ontario, Canada

**Keywords:** Venous thrombosis, thromboembolism, diagnosis, treatment, direct oral anticoagulants, aspirin, D-dimer, clinical prediction rule, factor XI inhibitors, advances

## Abstract

Focusing on the current state of the art, this article (a) describes recent advances in the understanding of the pathogenesis of venous thromboembolism (VTE), (b) discusses current approaches for the prevention, diagnosis and treatment of VTE, (c) outlines the role of aspirin for VTE prevention and treatment, and (d) highlights the unmet needs in VTE management and describes novel approaches to address them.

## Introduction

Venous thromboembolism (VTE), which includes deep vein thrombosis (DVT) and pulmonary embolism (PE), affects at least 1 in 1000 individuals annually and is the third most common cause of vascular death ^1^. About 1 in 20 patients with DVT and 1 in 10 with PE die within 30 days ^2^. In those who survive, long-term complications such as post-thrombotic syndrome (PTS) and chronic thromboembolic pulmonary hypertension are important causes of morbidity in up to 50% patients with DVT and 4% of those with PE, respectively ^3– 5^. About half a million VTE events occur each year in the US and more than half of these are related to hospitalization or institutionalization ^6^. Consequently, PE is the number one cause of preventable death in hospitalized patients.

Recent years have witnessed a renaissance in the diagnosis, prevention and treatment of VTE. Clinical prediction rules, D-dimer testing and non-invasive imaging modalities enable rapid, cost-effective and accurate diagnosis of VTE. Although anticoagulant therapy remains the mainstay for prevention and treatment of VTE, new findings have carved out a place for aspirin for VTE prevention, and treatment of VTE has been simplified and rendered safer by the introduction of the direct oral anticoagulants (DOACs) ^7^. By obviating the need for daily injection of parenteral anticoagulants such as low-molecular-weight heparin (LMWH) for initial VTE treatment and by circumventing the monitoring and dose adjustments required for vitamin K antagonists (VKAs) such as warfarin, DOACs enable outpatient treatment of most patients with DVT and many of those with PE, thereby reducing health-care costs.

However, despite advances in the diagnosis and treatment of VTE, problems persist. With increased reliance on imaging and widespread use of sensitive multi-detector computed tomography (CT) scanners, there is a potential for over-diagnosis of VTE ^8^. For example, extending the ultrasound examination to include veins below the knee results in more frequent diagnosis of calf DVT, the clinical significance of which is uncertain ^9^. Also uncertain is the optimal management of patients with CT-detected subsegmental PE, particularly those with a single subsegmental filling defect ^8^. Unnecessary anticoagulant treatment for such patients increases the risk of bleeding and adds to health-care costs. Finally, despite the safety advantages of the DOACs over VKAs, bleeding remains their major side effect. Therefore, there remains a need for safer anticoagulants ^10^.

Focusing on the current state of the art, this article (a) describes recent advances in the understanding of the pathogenesis of VTE, (b) discusses current approaches for the prevention, diagnosis and treatment of VTE, (c) outlines the role of aspirin for VTE prevention and treatment, and (d) highlights the unmet needs in VTE management and outlines novel approaches to address them.

## Advances in understanding the pathogenesis of venous thromboembolism

DVT usually starts in the deep veins of the calf where it forms in the valve pockets. Most calf DVTs resolve spontaneously. However, in about 20% of cases, calf DVT propagates into the more proximal veins where it produces pain and swelling of the leg by obstructing outflow of blood from the limb ^11^. These more proximal clots have the potential to break off and travel to the lungs to produce PE, which can be fatal. Advances in our understanding of the pathogenesis of VTE help to explain what causes VTE and why DVT usually starts in the valve pockets ^12^.

**Figure 1.**
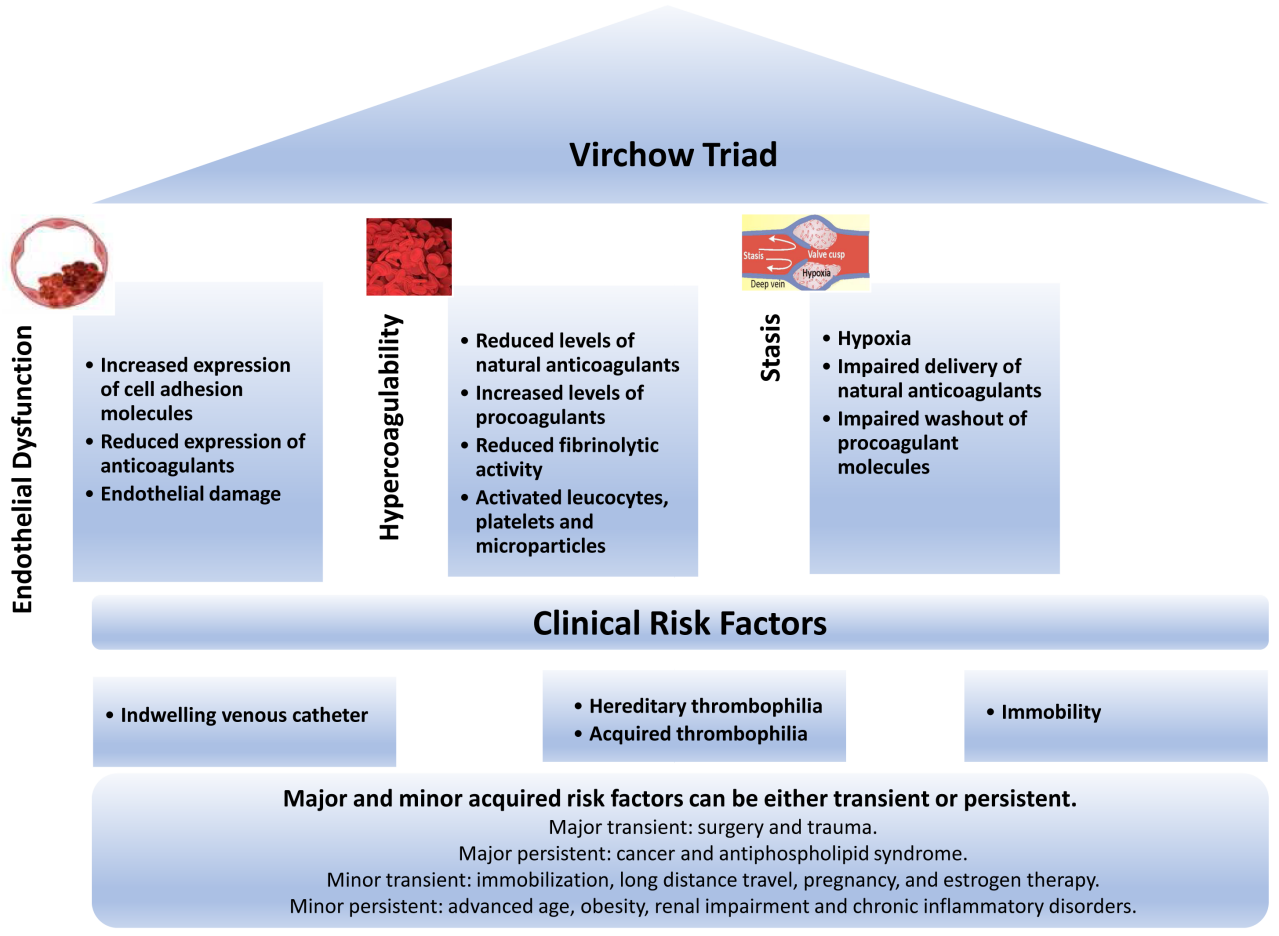
Pathogenesis of venous thromboembolism and risk factors.

The pathogenesis of VTE involves an interplay among hypercoagulability of the blood, sluggish blood flow, and damage or activation of the vessel wall – the components of the so-called Virchow triad ( Figure 1) ^13^. Hypercoagulability of the blood triggers a systemic increase in thrombin generation, whereas stasis and damage or activation of the vessel wall explain why DVT occurs more commonly in the deep veins of the leg than at other sites ^14^. Advances in our understanding of these processes are briefly described.

### Hypercoagulability of the blood

Many hereditary and acquired disorders are associated with hypercoagulability. Hereditary disorders that are potent risk factors for VTE include deficiencies of natural anticoagulants such as antithrombin, protein C and protein S ^15^. With insufficient amounts of these anticoagulants, there is increased thrombin generation and the potential for clot formation. Moderate genetic risk factors for VTE include factor (F) V Leiden and the prothrombin gene mutation. FV Leiden, an FV variant that is resistant to inactivation by activated protein C, is found in about 5% of individuals of European descent ^16^. The prothrombin gene mutation, which is found in about 3% of those of European descent, is a single-nucleotide polymorphism in the 3′ untranslated region that increases prothrombin synthesis ^17^. Delayed inactivation of FVa Leiden and elevated prothrombin levels lead to increased thrombin generation. Although these inherited thrombophilic disorders increase the risk of a first episode of VTE, their impact on the risk of recurrence is unclear. Consequently, routine thrombophilia testing is not recommended in patients with VTE since such tests rarely influence long-term management ^18^.

Acquired risk factors for VTE can be divided into those that are transient and those that are persistent ^19, 20^. Whether transient or persistent, risk factors can be major or minor. Examples of major transient risk factors are surgery and trauma, whereas minor transient risk factors include immobilization, long-distance travel, pregnancy, and estrogen therapy. Common examples of major persistent risk factors are cancer and antiphospholipid syndrome (APS), whereas minor persistent risk factors include advanced age, obesity, renal impairment, and chronic inflammatory disorders such as inflammatory bowel disease and rheumatoid arthritis ( Figure 1). Patients with both hereditary and acquired risk factors are at particularly high risk of VTE. Hereditary and acquired risk factors for VTE are associated with systemic activation of coagulation. Local factors explain why DVT usually starts in the calf.

### Venous thrombi originate in the valve pockets

Most venous thrombi originate in the valve pockets of the calf veins where there is stasis and hypoxia. Consistent with slow blood flow, contrast dye lingers in the valve pockets after venography ^20^. Hypoxia occurs because the valve leaflets are avascular and the oxygen tension in the valve pockets rapidly decreases when blood flow stops ^21^. Endothelial cells lining the valve pockets express antithrombotic proteins such as thrombomodulin and endothelial protein C receptor ^22^. Such expression is downregulated by local hypoxia or inflammation and by the loss of oscillatory shear-dependent transcription factors that endow perivalvular venous endothelial cells with an antithrombotic phenotype ^23^. Therefore, stasis and hypoxia in venous valve pockets promote a hypercoagulable microenvironment, which explains why DVT starts in this site.

Upper extremity DVT (UEDVT) is often triggered by central venous catheters or by extrinsic compression of the arm veins ^24^. Indwelling catheters or extrinsic compression retards blood flow, and local delivery of chemotherapy or antibiotics via the catheters can activate the endothelium. However, regardless of whether DVT occurs in the upper or lower extremities, the initiators of coagulation are likely to be similar.

### Initiators of coagulation in venous thromboembolism

Tissue factor initiates coagulation in patients with VTE, but the source of the tissue factor remains elusive ^25^. Overt damage to the vessel wall is rarely seen with DVT. However, hypoxia and inflammatory cytokines upregulate tissue factor expression by endothelial cells and circulating monocytes and induce endothelial cell expression of adhesion molecules such as P-selectin. The importance of endothelial cell tissue factor is uncertain, but P-selectin tethers tissue factor–bearing monocytes and microparticles onto their surface via interaction with P-selectin glycoprotein ligand 1 (PSGL-1) ^26^. By preventing such binding, P-selectin inhibitors attenuate venous thrombosis in animal models ^27^. Statins attenuate tissue factor expression by monocytes ^28^, which may explain why statins reduce the risk of recurrent VTE ^29, 30^. Like statins, proprotein convertase subtilisin/kexin type 9 (PCSK9) inhibitors reduce the risk of VTE and can attenuate monocyte function ^31– 33^. Therefore, adhesion of leukocytes and microparticles to the activated endothelium appears to be important in the pathogenesis of DVT.

**Figure 2.**
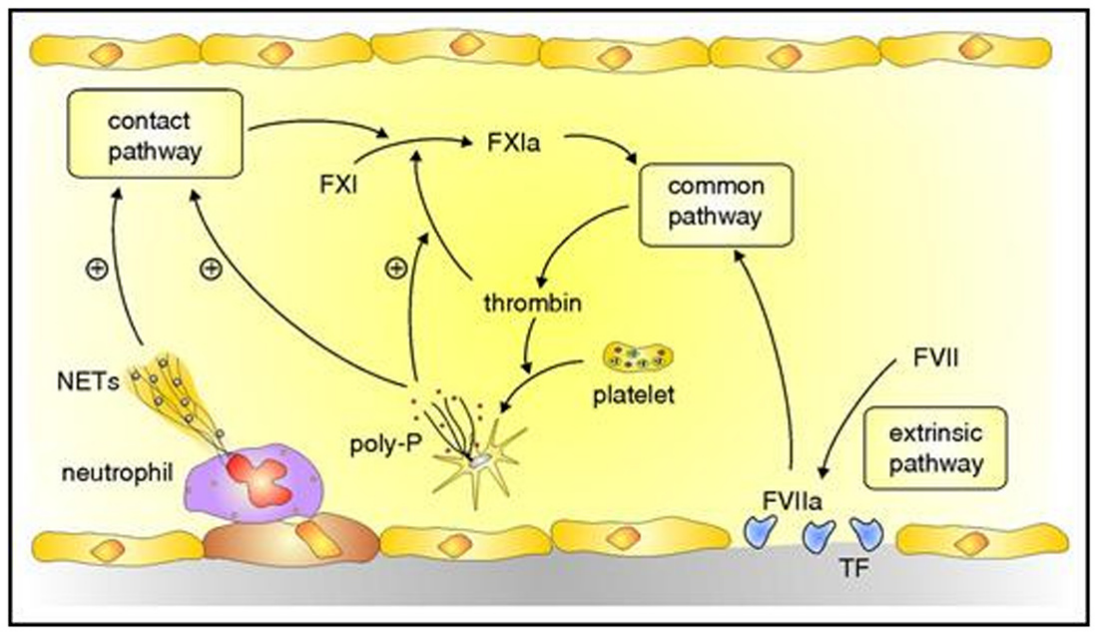
Initiators of coagulation. Coagulation is initiated by the extrinsic pathway when tissue factor (TF) exposed at sites of vascular injury binds and activates factor (F) VII. The FVIIa–TF complex activates FX in the common pathway to generate prothrombinase, which generates thrombin. Additional activation of coagulation occurs when thrombin-activated platelets release polyphosphate (poly-P) and activated neutrophils extrude DNA and RNA to form neutrophil extracellular traps (NETs). NETs and poly-P activate the contact pathway, which yields FXIa and leads to additional thrombin generation via the common pathway. Poly-P amplifies this pathway by promoting thrombin-mediated activation of FXI. Figure and legend reproduced with permission from the American Society of Hematology ^18^.

Emerging data suggest that, in addition to tissue factor, the contact system plays a part in the initiation of coagulation in VTE ( Figure 2) ^34– 36^. Neutrophil extracellular traps extruded from activated neutrophils trapped within venous thrombi provide a scaffold for platelet adhesion and activate FXII, thereby initiating coagulation via the intrinsic pathway ^37^. Likewise, inorganic polyphosphate released from activated platelets enhances FXII activation ^39^. Thrombin feedback activation of FXI, a reaction that is enhanced by polyphosphate, amplifies coagulation via the intrinsic pathway. With increasing evidence that the contact system is involved in the initiation of VTE, FXI has emerged as a target for new anticoagulants that may be safer than those that inhibit FXa or thrombin ^35, 36^.

### Role of platelets in venous thromboembolism

There is mounting evidence that platelets play a part in the pathogenesis of VTE. Venous thrombi consist of a central core that lies adjacent to the vessel wall and is rich in fibrin and trapped red blood cells and an overlying shell composed of platelet-rich layers ^40^. There is abundant evidence from mouse models that platelets are important in the initiation and propagation of venous thrombosis. Thus, P-selectin–dependent recruitment of platelets and leukocytes was essential for thrombus formation after incomplete ligation of the inferior vena cava ^41^. Inhibition or depletion of platelets or neutrophils suppressed thrombus formation in this model. Likewise, platelet depletion abrogated spontaneous venous thrombosis induced by concomitant knockdown of antithrombin and protein C. In addition, antiplatelet agents such as aspirin or clopidogrel attenuated thrombosis in a venous stasis model ^42, 43^.

Findings in mice may be translatable to humans because patients with acute VTE have evidence of systemic platelet activation and platelet-derived soluble P-selectin has been shown to be a biomarker for VTE in some studies ^44^. Therefore, platelets likely contribute to VTE, which explains why aspirin is effective for its prevention though less effective than anticoagulants ^46^.

### Stabilization, embolization and resolution of venous thrombi

In patients with DVT, the risk of PE appears to be dependent on the size and stability of the thrombus. The importance of thrombus size is highlighted by the fact that calf DVT rarely causes PE whereas up to 50% of patients with proximal DVT have associated PE. Clot stability also influences the risk of PE. Thrombin activates FXIII, which stabilizes the clot and renders it more resistant to lysis by cross-linking the fibrin polymers and by facilitating retention of red blood cells ^46, 47^. Enhanced thrombin generation and subsequent FXIII activation in patients with FV Leiden could explain the epidemiological evidence that DVT is more common than PE in such patients. This concept is supported by the results of studies in mice ^48^. Thus, treatment with dabigatran after ferric chloride induced femoral vein thrombosis enhanced PE by suppressing thrombin-mediated FXIII activation. Conversely, FXIII supplementation reduced the risk of PE without promoting thrombus growth.

Thrombus resolution depends on fibrinolysis, which occurs when plasminogen is converted to plasmin. Degradation of thrombi by plasmin likely explains why most calf DVTs resolve spontaneously. Migration of leukocytes into the thrombus promotes fibrinolysis and contributes to early thrombus resolution by releasing clot-digesting enzymes ^49^. Impaired fibrinolysis has been linked to recurrent VTE, and up to 50% of patients with proximal DVT have ultrasound evidence of residual vein occlusion 1 year after their index event. Reduced blood flow contributes to PTS, which is the major long-term complication of DVT.

## Advances in diagnosis of venous thromboembolism

In patients with suspected VTE, the goal of diagnosis is to rapidly and accurately distinguish those with the condition from those without it. This is essential because patients with VTE require rapid initiation of anticoagulant therapy, whereas those without the condition do not. The wrong diagnosis is problematic because failure to prescribe anticoagulant therapy to patients with VTE can enable thrombus extension and fatal PE and because inappropriate administration of anticoagulants to those without VTE can lead to fatal bleeding. Therefore, rapid and accurate diagnosis is essential.

Diagnosing VTE on the basis of clinical signs and symptoms alone is unreliable, so non-invasive imaging tests are often needed. Compression ultrasound is used for the diagnosis of DVT and CT pulmonary angiography; less frequently, ventilation-perfusion (VQ) lung scanning is used for the diagnosis of PE. The challenge with the reliance on imaging for ruling out VTE is that most patients with suspected VTE do not have it. The diagnosis is confirmed in only about 20% and 5%, respectively, of those with suspected DVT and PE ^50, 51^. With an estimated annual incidence of VTE of about 0.5 million in the US, this would translate to at least 2 million patients undergoing diagnostic imaging for suspected VTE each year. Relying on imaging in such a large number of patients is problematic because of the cost to health-care systems, the unnecessary exposure to radiation for the majority of patients with suspected PE, and the potential for over-diagnosis of events of uncertain clinical significance, such as calf DVT and subsegmental PE.

**Figure 3.**
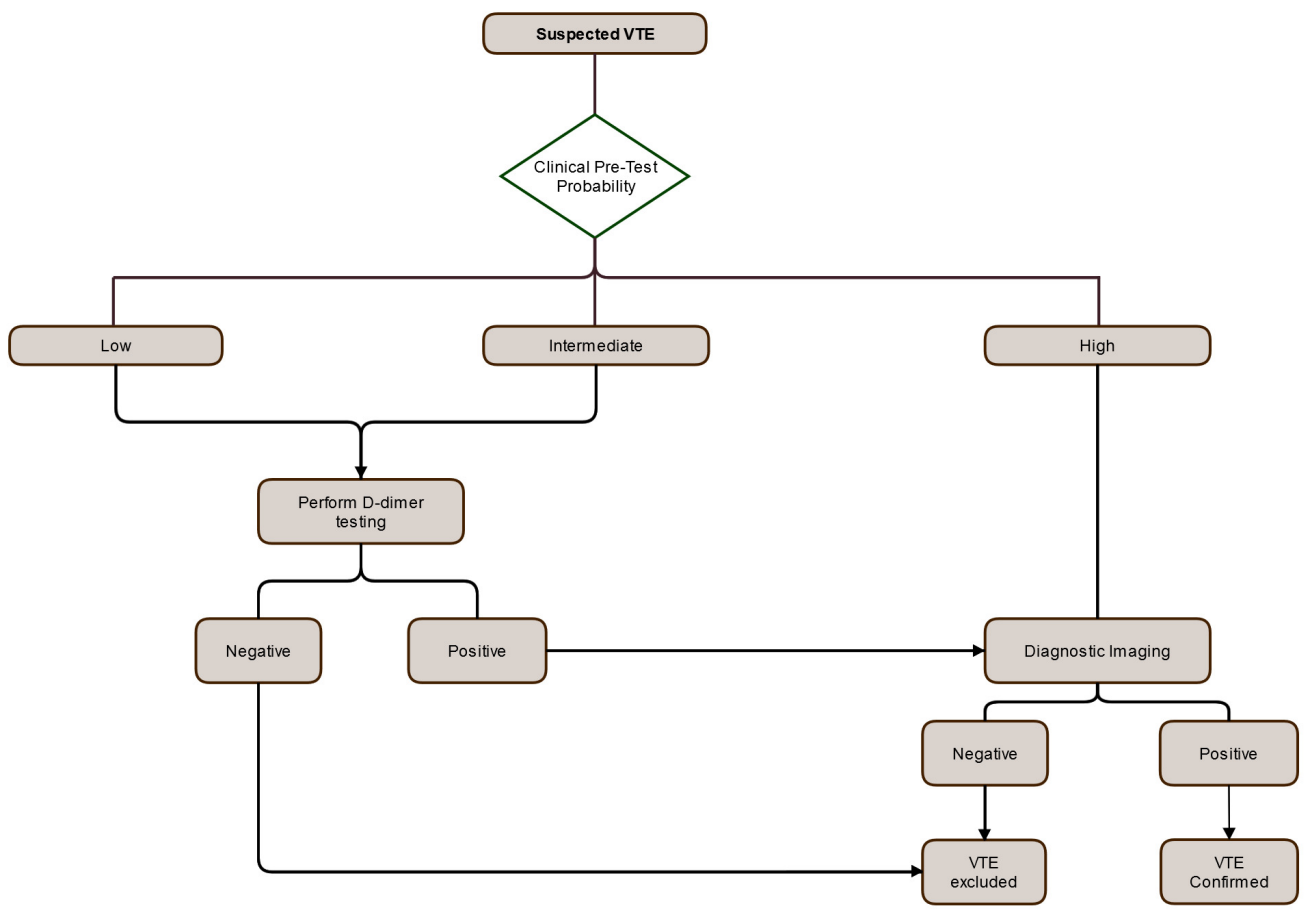
Approach to venous thromboembolism (VTE) diagnosis using pre-test probability, D-dimer and diagnostic imaging. Diagnostic algorithm in patients with suspected deep vein thrombosis (DVT) or pulmonary embolism (PE). The first step is assessment of the clinical pretest probability. Patients with a low or intermediate pretest probability should undergo D-dimer testing. A negative D-dimer excludes the diagnosis, whereas those with a positive D-dimer test should undergo diagnostic imaging with compression ultrasound and computed tomography pulmonary angiography to rule out DVT and PE, respectively. Patients with a high pretest probability require imaging.

The diagnosis of VTE has been streamlined with the development of reliable clinical prediction rules to determine the pretest probability of VTE and the introduction of sensitive and rapid assays for D-dimer. The first advance in diagnosis started in the 1990s when Wells and colleagues derived clinical prediction rules that could reproducibly classify patients with suspected VTE into low, intermediate, and high pretest probability categories on the basis of a combination of symptoms and signs and the absence or presence of risk factors for VTE and an alternative diagnosis ( Figure 3) ^51, 52^. Patients with a high pretest probability of VTE require imaging for confirmation. The risk of VTE in patients with a low pretest probability is not low enough to obviate the need for diagnostic imaging. This problem was overcome by combining the pretest probability with a sensitive assay for measurement of the level of D-dimer in plasma ^54^. D-dimer is a plasmin-derived breakdown product of cross-linked fibrin. D-dimer levels are elevated in most patients with VTE. Therefore, the combination of a low or intermediate pretest probability of VTE with a negative D-dimer test, defined as a D-dimer level below 500 μg/L, effectively rules out VTE. In contrast, imaging is needed to rule out VTE in patients with a low or intermediate pretest probability and a positive D-dimer test ( Figure 3).

Two more recent advances have further reduced the need for diagnostic imaging in specific settings: the Pulmonary Embolism Rule-Out Criteria (PERC) ^55, 56^, which are used to obviate the need for further investigation for PE in low-risk patients presenting to the emergency department, and optimization of the D-dimer cut-off to enhance specificity without compromising sensitivity in outpatients with suspected VTE ^57– 59^.

### Pulmonary Embolism Rule-Out Criteria

The PERC rule consists of eight clinical variables: age over 50 years, heart rate over 100 beats per minute, oxygen saturation less than 95%, unilateral leg swelling, hemoptysis, recent surgery or trauma, prior VTE, and estrogen use ^55, 56^. In patients with a low pretest probability of PE and none of these variables, PE can be safely excluded without the need for D-dimer determination or other diagnostic testing. The PERC rule cannot be applied to patients with a moderate or high pretest probability of PE or to those with a PERC score of 1 or more. Such patients require further evaluation with D-dimer testing or diagnostic imaging. The PERC rule, which has been validated in over 14,000 patients with suspected PE, has a sensitivity of 97% and a specificity of 22% in the emergency department settings where the prevalence of PE is below 15% ^60^. With the availability of large databases and advances in computing and analytical techniques, emerging data suggest that machine-based algorithms may be superior to traditional clinical prediction rules and, at least in one study, could have obviated the need for ultrasound imaging in up to 54% of patients with suspected DVT while maintaining a low false-negative rate (<1%) ^61^.

### Adjusted D-dimer cut-off

Adjustment of the D-dimer cut-off is another maneuver that can be used to reduce the need for diagnostic imaging. Thus, instead of using a fixed cut-off of 500 µg/L, the D-dimer cut-off can be adjusted on the basis of patient age or pretest probability of VTE ^57– 59^. For age adjustment, the cut-off is set at 10 times the patient’s age for those over 50 years of age (for example, cut-off of 10 × 70 = 700 µg/L for a person 70 years of age) ^57^. Such adjustment reduces the rate of false positives because D-dimer levels increase with age. In outpatients 75 years of age or older with a low to moderate pretest probability of PE, use of an age-adjusted cut-off instead of a fixed cut-off increased the proportion of patients in whom PE could be excluded on the basis of D-dimer alone from 6.4 to 29.7% in one study ^57^. Therefore, use of an age-adjusted D-dimer cut-off for evaluation of PE in outpatients may improve quality of care and reduce health-care costs in older patients.

To reduce the need for diagnostic imaging, the D-dimer cut-off can also be adjusted on the basis of pretest probability. For this approach, a cut-off of 500 µg/L is retained for patients with an intermediate pretest probability, but the cut-off is increased to 1000 µg/L for those with a low pretest probability. In patients with suspected DVT or PE, use of a graduated D-dimer cut-off has been shown to reduce the need for diagnostic imaging ^58, 59, 62^.

A graduated D-dimer cut-off has also been used for the diagnosis of PE in pregnancy ^63^. Pregnant women are first assessed for three clinical criteria: clinical signs of DVT, hemoptysis, and the absence or presence of an alternative diagnosis. In those with no criteria, a cut-off of 1000 µg/L is used whereas a 500 µg/L cut-off is used if any criterion is present. When evaluated in 510 pregnant women with suspected PE, this diagnostic approach ruled out PE without the need for imaging in 39% of patients ^63^.

## Uncertainties in venous thromboembolism diagnosis

Despite recent advances in VTE diagnosis, uncertainties persist. These include the diagnosis of UEDVT and recurrent DVT.

### Upper extremity deep vein thrombosis

UEDVT accounts for about 5% of all DVT and occurs mainly in patients with central venous catheters or secondary to thoracic outlet syndrome, the so-called Paget–Schroetter syndrome, which typically occurs in the dominant arm of young athletes. Diagnosis of UEDVT currently relies on compression ultrasound imaging. Use of a clinical prediction rule based on clinical signs and presence or absence of risk factors or use of D-dimer alone cannot safely rule out UEDVT. Instead, a combined strategy is needed to enhance diagnostic performance. In a multi-centre prospective management study that enrolled 406 patients with suspected UEDVT, the combination of a low pretest probability and a normal D-dimer level safely excluded UEDVT in 21% of patients without the need for ultrasonography ^64^. However, the combination was less efficient for ruling out UEDVT in patients with central venous catheters or with cancer and in those who were hospitalized; this was possibly due to the poor specificity of D-dimer in such patients ^65^. Additional studies are needed to validate the performance of this diagnostic strategy in various populations.

### Recurrent deep vein thrombosis

The diagnosis of recurrent DVT is challenging because a history of DVT increases the pretest probability in patients with suspected DVT and reduces the specificity of D-dimer. However, it appears that a low pretest probability and a normal D-dimer level safely exclude recurrent DVT in patients not taking anticoagulants ^66^. Prior DVT reduces the diagnostic specificity of ultrasonography because residual ultrasound abnormalities are found in at least 50% of patients 1 year after their index event. Consequently, compression ultrasonography is useful only if comparison with a previous examination shows involvement of a new venous segment.

Preliminary studies suggest that 18F-fluorodeoxyglucose positron emission tomography/CT (FDG-PET/CT) and magnetic resonance direct thrombus imaging (MRDTI) may be useful for the diagnosis of recurrent DVT. FDG-PET/CT has the potential to differentiate new thrombus from old thrombus because of the preferential uptake of fluorodeoxyglucose by the inflammatory cells that accumulate in newly formed clots ^67, 68^, whereas MRDTI detects the paramagnetic signal induced by release of methemoglobin from fresh thrombus ^69, 70^. In a small prospective study that included 39 patients with recurrent DVT confirmed by the presence of a new non-compressible venous segment on ultrasonography and 42 asymptomatic patients with residual vein occlusion after an index DVT at least 6 months earlier, MDRTI had 95% sensitivity and 100% specificity for detecting recurrent DVT ^70^. These promising results require confirmation, but, even so, the limited availability of MDRTI may reduce its utility.

## Prevention and treatment of venous thromboembolism

The DOACs are licensed for thromboprophylaxis after elective hip or knee arthroplasty and for initial, long-term and extended VTE treatment. In addition, betrixaban and rivaroxaban are approved in the US for thromboprophylaxis in medically ill patients at high risk for VTE. Because they can be given orally in fixed doses without coagulation monitoring, the DOACs simplify treatment and enhance adherence and persistence. In the next section, we summarize the results of the phase 3 trials that prompted regulatory approval of the DOACs for VTE prevention and treatment and we highlight the recent developments that have led to their expanded use for prevention and treatment of cancer-associated thrombosis.

### Direct oral anticoagulants for primary venous thromboembolism prevention

More than half of the 0.5 million VTE events that occur each year in the US are related to recent hospitalization or institutionalization, and up to 75% of all fatal PEs are associated with recent hospitalization. Therefore, VTE is the number one cause of preventable death in hospitalized patients. Traditionally, LMWH was used for thromboprophylaxis, but the need for daily subcutaneous injections is burdensome for many patients. Consequently, the DOACs are an attractive alternative.

***Hip and knee arthroplasty*.** Among surgical patients, those undergoing elective hip or knee arthroplasty are at highest risk for VTE and the risk persists for several weeks after surgery. In phase 3 trials that included over 35,000 patients, the DOACs (dabigatran, rivaroxaban and apixaban) were at least as effective as LMWH for prevention of symptomatic VTE and were not associated with an increased risk of major bleeding ^71^. Based on these results, the DOACs are licensed for initial and extended thromboprophylaxis in patients undergoing elective hip or knee arthroplasty in several jurisdictions and are recommended by various guidelines ^72^.

With improvements in perioperative care and the gradual reduction in mortality rates (including fatal VTE) after elective major orthopedic surgery ^73^, recent trials are re-examining the role of extended DOAC thromboprophylaxis. In the EPCAT-II trial, which included 3424 patients undergoing elective hip or knee arthroplasty who were given an initial 5-day course of rivaroxaban, extended thromboprophylaxis with low-dose aspirin for up to 30 days was non-inferior to extended prophylaxis with rivaroxaban for the prevention of symptomatic VTE (0.64% vs. 0.70%) and rates of major bleeding with aspirin and rivaroxaban were similar (0.47% vs. 0.29%) ^74^. The most recent guidelines suggest either aspirin or an anticoagulant (DOAC or LMWH) for extended thromboprophylaxis after elective hip or knee arthroplasty ^74^. Ongoing trials (PEPPER and EPCAT-3; ClinicalTrials.gov Identifiers NCT02810704 and NCT04075240, respectively) are examining whether upfront aspirin is non-inferior to anticoagulant prophylaxis.

***Medically ill*.** Whereas VTE is frequent after major orthopedic surgery, the rate of VTE in hospitalized medical patients is lower. Consequently, practice has shifted from routine thromboprophylaxis to a risk-adapted approach whereby thromboprophylaxis is offered only to high-risk hospitalized medical patients identified by using risk assessment models or by an elevated D-dimer level. In three trials that collectively included 22,142 hospitalized medical patients deemed to be at high risk for VTE, extended thromboprophylaxis with 30 to 42 days of a DOAC (apixaban, betrixaban or rivaroxaban) was compared with a 10 ± 4–day course of enoxaparin. In a meta-analysis of these trials, the efficacy of DOACs and LMWH was similar during the initial 10 ± 4–day period, but there was more bleeding with the DOACs. Extended thromboprophylaxis with DOACs was no more effective in reducing the risk of symptomatic VTE compared with enoxaparin followed by placebo, but it was associated with more major bleeding ^75^. The MARINER trial, which included 12,019 patients, evaluated a risk stratification strategy performed at the time of hospital discharge to identify medical patients at high risk of VTE who might benefit from a 45-day course of rivaroxaban. Although rivaroxaban was not superior to placebo for preventing the primary efficacy outcome, the composite of symptomatic VTE and VTE-related mortality (0.83% vs. 1.10%), rivaroxaban significantly reduced the risk of symptomatic VTE from 0.42% to 0.18% ^76^. Rates of major bleeding were low, but the rate was higher with rivaroxaban than with placebo (0.28% vs. 0.18%) – a difference that was not statistically significant. Overall, therefore, the net clinical benefit of extended DOAC thromboprophylaxis over a shorter duration of thromboprophylaxis with LMWH is limited in medically ill patients.

Despite being licensed for thromboprophylaxis in high-risk medically ill patients in the US, betrixaban and rivaroxaban are not approved in other jurisdictions. Furthermore, the recent American Society of Hematology guidelines recommend LMWH over DOACs for thromboprophylaxis in hospitalized medically ill patients and do not recommend routine extension of thromboprophylaxis after hospital discharge ^75^.

***High-risk cancer patients*.** Recent data in selected ambulatory patients undergoing cancer therapy suggest that extended DOAC prophylaxis may be of benefit. Thus, rivaroxaban and apixaban were compared with placebo in cancer patients identified as being at risk of VTE by a Khorana score of 2 or higher ^77, 78^. The Khorana score predicts the risk of thrombosis in patients undergoing treatment for cancer on the basis of the type of cancer, body mass index, and blood counts. A score over 2 is associated with a VTE risk of at least 6% at 3 months. In the CASSINI trial, rivaroxaban was compared with placebo in 841 such patients. Compared with placebo, rivaroxaban prophylaxis for up to 180 days resulted in less VTE (6.0% vs. 8.8%) without a significant increase in major bleeding (2.0% vs 1.0%) but 44% of patients discontinued study drug during the trial. In the on-treatment analysis, rivaroxaban significantly reduced VTE by about 60% (2.6% vs. 6.4%) ^77^. In the AVERT trial, which compared apixaban with placebo in 574 patients with cancer, apixaban significantly reduced the risk of VTE by about 60% (4.2% vs. 10.2%) without a significant increase in major bleeding during the on-treatment period (2.1% vs 1.1%) ^78^. These results have the potential to change practice, and recent guidelines suggest primary prophylaxis with rivaroxaban or apixaban for up to 6 months in ambulatory patients undergoing cancer therapy who are deemed at high risk of VTE and have no risk factors for bleeding ^79^.

### Direct oral anticoagulants for treatment of venous thromboembolism

Until recently, patients with acute VTE required initial treatment with a rapidly acting parenteral anticoagulant (usually LMWH) overlapped and followed by a VKA such as warfarin. Though safe and effective, such treatment was inconvenient for patients and costly for health-care systems because LMWH requires daily subcutaneous injection, and warfarin requires frequent coagulation monitoring and dose adjustments to ensure that the international normalized ratio (INR) is therapeutic. Given in fixed doses without routine laboratory monitoring, DOACs have streamlined VTE treatment.

***Treatment of acute venous thromboembolism in patients without active cancer*.** Dabigatran, rivaroxaban, apixaban and edoxaban were compared with LMWH/VKA for the initial and long-term treatment of acute VTE in phase 3 trials that collectively enrolled 23,750 patients. In a meta-analysis of these trials, the DOACs were non-inferior to LMWH/VKA therapy for reducing the risk of recurrent VTE and were associated with a 40% reduction in the risk of major bleeding ^80^.

***Extended venous thromboembolism treatment in patients without active cancer*.** The efficacy and safety of the DOACs for extended VTE treatment have been examined in five trials, which included patients who had completed anticoagulation for their index event. In the placebo-controlled trials, full-dose treatment with apixaban, dabigatran or rivaroxaban reduced the risk of VTE by at least 80% but increased the risk of clinically relevant bleeding (the composite of major and clinically relevant non-major bleeding) by about two-fold ^81– 83^. In the active controlled RE-MEDY trial, dabigatran was non-inferior to warfarin and was associated with an almost 50% reduction in clinically relevant bleeding ^81^. Although edoxaban was not evaluated in a dedicated extension trial, in a sub-analysis of the HOKUSAI VTE trial, edoxaban was non-inferior to warfarin and was associated with similar rates of clinically relevant bleeding in patients who received 3 to 12 months of anticoagulation ^84^. Consequently, when used for extended treatment, DOACs reduce the risk of recurrence by over 80% and are associated with more bleeding than placebo but appear to be safer than warfarin.

For extended VTE treatment, the efficacy and safety of low-dose and full-dose regimens of apixaban or rivaroxaban were compared in two trials. In a meta-analysis of these trials, the low-dose regimens were associated with similarly low rates of recurrent VTE as the high doses and a trend for lower rates of clinically relevant bleeding ^85^. These findings contrast with what was found with low-intensity warfarin (target INR of 1.5 to 2.0), which was less effective than usual-intensity warfarin (target INR of 2.0 to 3.0) for prevention of recurrent VTE ^86^. Consequently, lower-dose regimens of rivaroxaban or apixaban likely provide a more favorable benefit–risk profile than full-dose regimens.

Finally, aspirin was compared with placebo for secondary VTE prevention in 1224 patients with unprovoked VTE who had completed at least 3 months of anticoagulant therapy for their index event ^87^. Compared with placebo, aspirin reduced the rate of recurrent VTE by 32% from 7.5 to 5.1% and the rates of major bleeding with placebo and aspirin were similar (0.4% and 0.5%, respectively). However, when aspirin was compared with the usual or the lower dose of rivaroxaban for prevention of recurrent VTE, both doses of rivaroxaban reduced the rate of recurrent VTE by 70% without significantly increasing the risk of major bleeding ^88^. Therefore, DOACs are more effective than aspirin for extended VTE treatment.

With a better benefit–risk profile than enoxaparin/VKA treatment and the convenience of fixed oral dosing without the need for monitoring, DOACs are now given preference in the guidelines over VKAs for initial, long-term and extended VTE treatment in patients without active cancer. Lower-dose regimens of apixaban or rivaroxaban appear to have a better benefit–risk profile than the usual-dose regimens for extended VTE treatment. Consequently, dose reduction can be considered for most VTE patients who require extended treatment. However, it remains unclear whether the low-dose regimens are as effective as the high-dose regimens in patients at high risk of recurrence, such as those with metastatic cancer or high-risk thrombophilia, because few such patients were included in the trials.

***Venous thromboembolism treatment in patients with active cancer*.** Until recently, LMWH was recommended over VKAs or DOACs for the treatment of cancer-associated VTE because the initial phase 3 trials that compared the DOACs with VKAs either excluded patients with cancer or included only a limited number of them. This changed with the results of more recent trials ( Table 1). The HOKUSAI VTE Cancer trial compared the efficacy and safety of edoxaban and dalteparin in 1046 patients with cancer-associated VTE ^89^. Edoxaban was non-inferior to dalteparin for the composite primary efficacy outcome of recurrent VTE and major bleeding. In further analyses, edoxaban was associated with a lower rate of first recurrent VTE and a significantly higher rate of major bleeding, mostly driven by an excess in upper gastrointestinal bleeds. Two smaller studies with rivaroxaban and apixaban yielded similar results. In the Select-d trial, which included 406 patients with cancer-associated VTE, rivaroxaban significantly reduced the risk of recurrent VTE but was associated with more major and clinically relevant non-major bleeding, most of which was gastrointestinal in origin ^90^. In the ADAM VTE study, apixaban was compared with dalteparin in 300 patients ^91^. Although apixaban significantly reduced the risk of recurrent VTE with similar rates of clinically relevant bleeding, the trial was underpowered to assess differences in major bleeding. The larger CARAVAGGIO trial compared apixaban with dalteparin in 1170 patients with cancer-associated VTE ^92^. Rates of recurrent VTE with apixaban and dalteparin were 5.6% and 7.9%, respectively, whereas rates of major bleeding were 3.8% and 4.0%, respectively, and rates of clinically relevant non-major bleeding were 9.0% and 6.0%, respectively. Although rates of major gastrointestinal bleeding with apixaban and dalteparin were similar (1.9% and 1.7%, respectively), less than 5% of the patients included in the study had cancers of the upper gastrointestinal tract, which were the cancers most frequently associated with major bleeding in the trials with edoxaban and rivaroxaban ( Table 1). Therefore, taken together, these trials indicate that the DOACs are at least as effective as dalteparin for the prevention of recurrence in VTE patients with active cancer, but the DOACs are associated with more bleeding in patients with gastrointestinal cancers, particularly in those with intact primary tumors.

**Table 1.** Efficacy and safety of direct oral anticoagulants for treatment of cancer-associated venous thromboembolism.

Studies ^81– 84^	Intervention	Control	Primary outcome	Symptomatic VTE	Bleeding	Mortality
HOKUSAI VTE Cancer (n = 1050)	Edoxaban ^a^	Dalteparin ^b^	VTE or MB at 12 months 12.8% vs. 13.5% HR 0.97 (0.70–1.36)	7.9% vs. 11.3% HR 0.71 (0.48–1.06)	MB: 6.9% vs. 4.0% HR 1.77 (1.03–3.04) Major GI bleeding: 3.8% vs. 1.1%	39.5% vs. 36.6% HR 1.12 (0.92–1.37)
SELECT-D ^c^ (n = 406)	Rivaroxaban ^d^	Dalteparin ^b^	VTE at 6 months	4% vs. 11% HR 0.43 (0.19–0.99)	6% vs. 4% HR 1.83 (0.68–4.96) Major GI bleeding: 3.9% vs. 2.0%	25% vs. 30%
ADAM VTE ^e^ (n = 300)	Apixaban ^f^	Dalteparin ^b^	MB at 6 months	0.7% vs. 6.3% HR 0.09 (0.013–0.78)	0% vs. 1.4% Major GI bleeding: none	16% vs. 11%
CARAVAGGIO (n = 1170)	Apixabanf	Dalteparinb	VTE at 6 months	5.6% vs. 7.9% HR 0.63 (0.37–1.07)	3.8 vs 4.0% HR 0.82 (0.40–1.69) Major GI bleeding: 1.9% vs. 1.7% HR 1.05 (0.44–2.50)	23.4% vs. 26.4% HR 0.82 (0.62–1.09)

GI, gastrointestinal; HR, hazard ratio (with 95% confidence interval in parentheses); MB, major bleeding; n, number of patients who underwent randomization; VTE, (objectively diagnosed) venous thromboembolism. ^a^Lead-in of therapeutic low-molecular-weight heparin for at least 5 days followed by edoxaban 60 mg once daily or 30 mg once daily in patients with creatinine clearance of 30 to 50 mL/min, body weight of less than 60 kg, or receiving concomitant strong P-glycoprotein inhibitors. ^b^200 IU/kg daily for 30 days followed by 150 IU/kg daily thereafter. ^c^Because of slow recruitment, sample size was reduced to 406 from planned 530 patients. ^d^15 mg twice daily for 21 days followed by 20 mg once daily thereafter. ^e^With a sample size of 300 patients, this study was powered to detect only large (>78%) reductions in the primary outcome. Proportions of patients with upper GI cancers were generally low in these trials: HOKUSAI VTE Cancer (5.1%), SELECT‐D (7.3%), ADAM VTE (3.6%) and CARAVAGGIO (4.6%). ^f^10 mg twice daily for 7 days followed by 5 mg twice daily thereafter.

The favorable results of these trials have prompted changes in the guidelines ^93^. DOACs or LMWH are now recommended for the treatment of cancer-associated VTE except for patients at higher risk of gastrointestinal or genitourinary bleeding, in whom LMWH is still preferred.

## Contraindications to direct oral anticoagulants

DOACs are contraindicated in high-risk patients with APS because two trials showed more thromboembolic events with rivaroxaban than with VKAs. Thus, the TRAPS study compared rivaroxaban with warfarin in 120 APS patients who were triple-positive for lupus anticoagulant and anti-cardiolipin and anti-β2-glycoprotein I antibodies ^94^. The study was stopped early because there were more venous and arterial thromboembolic events and major bleeding events with rivaroxaban than with warfarin. A second study comparing rivaroxaban with VKAs in 190 patients with APS revealed higher rates of recurrent thrombosis with rivaroxaban than with VKAs (11.6% and 6.3%, respectively), including nine strokes in the rivaroxaban group and none with VKAs. Rates of major bleeding with rivaroxaban and VKAs were similar (6.3% and 7.4%, respectively) ^95^. Therefore, the DOACs are contraindicated in high-risk APS patients and such patients require treatment with VKAs. The DOACs are small molecules that can pass through the placenta and into breast milk. Consequently, they are contraindicated in pregnancy and in nursing mothers ^96^.

## Conclusions and future directions

Advances in VTE diagnostic pathways are reducing the need for diagnostic imaging, thereby addressing the issues associated with relying on imaging in a large number of patients: cost to health care, unnecessary exposure to radiation for the majority of patients with suspected PE, and potential for over-diagnosis and over-treatment of events of uncertain clinical significance, such as calf DVT and subsegmental PE. Additional studies are required to examine the safety of withholding anticoagulation in patients with subsegmental PE, particularly those with only a single filling defect ^8^.

The DOACs have transformed the prevention and treatment of VTE by eliminating the need for subcutaneous injections of LMWH or for coagulation monitoring and dose adjustment for VKAs. Nonetheless, bleeding remains the major complication of anticoagulant therapy. Although the rates of bleeding are lower with DOACs than with VKAs, even with the DOACs, the 3-month rate of major bleeding is about 3.3% in clinical practice and is even higher in older patients and in those with chronic kidney disease or cancer ^97^. Therefore, there remains a need for safer anticoagulants.

Epidemiological data and studies in animals provide increasing evidence that FXI is essential for thrombus stabilization and growth and less important for hemostasis ^10^. Consequently, FXI has emerged as a target for the development of safer anticoagulants. Such anticoagulants include an antisense oligonucleotide that reduces FXI levels by blocking its synthesis in the liver, antibodies that inhibit FXIa or that bind to FXI and block its activation, and small molecules that bind to the active site of FXIa and block its activity. Phase 2 studies with the FXI antisense oligonucleotide and with osocimab, a FXIa-directed inhibitory antibody, have been reported ^35, 36^. When compared with enoxaparin for thromboprophylaxis in patients undergoing elective knee arthroplasty, both agents were effective and were associated with low rates of bleeding. Ongoing studies are evaluating these and other FXI inhibitors for various indications ( Table 2) ^98^. If the favorable results observed in phase 2 are confirmed in phase 3 trials, FXIa inhibitors may provide new opportunities to reduce the burden of VTE and bleeding.

**Table 2.** Strategies to inhibit factor XI.

Agents	Mechanisms of action of FXI inhibitors
Antisense oligonucleotides (ASO)	Reduce hepatic synthesis of FXI by inducing catalytic degradation of FXI mRNA (for example, IONIS-FXI Rx) ^a^
Monoclonal antibodies	Suppress FXIa generation and/or inhibit FXIa activity (for example, BAY1213790 (osocimab) ^b^, MAA868 (abelacimab), AB023)
Small molecules	Bind to catalytic domain (for example, JNJ-70033093, BAY2433334, EP-7041, ONO-5450598)
Aptamers	Bind to FXI and/or FXIa and block its activity

^a^FXI ASO for prevention of venous thromboembolism (evaluated in a phase 2 randomized controlled trial: NCT01717761). ^b^Osocimab (evaluated in a phase 2 randomized, active-comparator-controlled, multi-center study (NCT03276143) to assess the safety and efficacy of different doses of BAY1213790 for the Prevention of Venous Thromboembolism in Patients Undergoing Elective Primary Total Knee Arthroplasty, Open-label to Treatment and Observer-blinded to BAY1213790 Doses (FOXTROT) ^36^.
